# Significance of chondrocyte viability in postmortem interval assessments and chondrocyte viability assay

**DOI:** 10.1007/s00414-025-03549-4

**Published:** 2025-07-16

**Authors:** Anita Galić Mihić, Davor Mayer, Katerina Jazbec Gradišar, Elvira Maličev, Rok Blagus, Pero Hrabač, Armin Alibegović

**Affiliations:** 1 Institute of Forensic Medicine and Criminalistics, School of Medicine, University of Zagreb, Šalata 11, Zagreb, 10000 Croatia; 2 Slovenian Institute for Transfusion Medicine, Šlajmerjeva 6, Ljubljana, 1000 Slovenia; 3 Institute of Biostatistics and Medical Informatics, Faculty of Medicine, University of Ljubljana, Vrazov trg 2, Ljubljana, 1000 Slovenia; 4 Faculty of Mathematics, Natural Sciences and Information Technologies, University of Primorska, Glagoljaška 8, Koper, 6000 Slovenia; 5 IT Department at School of Medicine, University of Zagreb, Šalata 3, Zagreb, 10000 Croatia; 6 Institute of Forensic Medicine, Faculty of Medicine, University of Ljubljana, Korytkova 2, Ljubljana, 1000 Slovenia

**Keywords:** Knee cartilage, Chondrocyte, Time since death, Flow cytometry, Cell viability analyzer

## Abstract

Determination of the postmortem interval (PMI) is one of the most challenging problems in forensic medicine. The main aim of this study was to determine the dynamics of the decrease in the fraction of viable chondrocytes excluded from the donors’ knees for PMI determination. The other aim was to find an appropriate method for chondrocyte viability assay. We analyzed osteochondral cylinders from 35 donors (28 males and 7 females), aged 44 to 90 years, whose bodies were stored in refrigerators at temperature of 8 ± 2 °C, during the period from 4 to 83 days postmortem. The proportion of viable chondrocytes was determined by flow cytometry (FC) and cell viability analyzer (CVA). For FC we used RedDot™1 to mark all chondrocytes with nuclei (method FCN) and 7-AAD, to distinguish live/dead cells (method FCC) among RedDot™1 positive cells. Results revealed that chondrocytes from the knee cartilage can be found alive after more than two months postmortem. We observed that even in controlled temperatures and environment, the predicted interval for PMI is too wide for this method to be used in daily forensic practice, likely due to the relatively small number of donors considered in our study, and other unknown factors that affect the viability of chondrocytes in dead bodies. This could be verified with a larger number of donors followed over a longer period. FCC is a slightly superior method over CVA and FCN in terms of its ability for PMI determination.

**Supplementary Information:**

The online version contains supplementary material available at 10.1007/s00414-025-03549-4.

## Introduction

Estimation of the postmortem interval (PMI) forms a crucial component for all forensic pathologists [1]. It is not only important in criminal cases but also in civil cases where they have legal implications stemming from insurance and inheritance obligations [2, 3]. The main objectives for estimating PMI are to give a primary idea of the time of assault to the police and to check whether it is reliable with the alibi of the suspect [4].

Various methods are used to estimate the time since death. The gold standard for death time estimation is a previously established nomogram method based on the two-exponential model of body cooling by Henssge. To reduce the margin of error of the nomogram method, compound methods were developed based on electrical and mechanical excitability of skeletal muscle, pharmacological excitability of the iris, rigor mortis, postmortem lividity, along with some novel methods such as the analysis of vitreous potassium and hypoxanthine, and the analysis of biological clock genes [5, 6]. Unfortunately, the above indicators can only be applied within 48–72 h after death and no longer [7]. When decomposition starts to progress, existing methods are unusable and accurate estimation of PMI is very complex, if not impossible [8].

In order to find a reliable and objective method for assessing PMI from severely decomposed bodies, many techniques have been studied. Reliability can only be provided empirically by statistical analysis in field studies. Determining PMI requires the calculation of measurable data along a time-dependent curve back to the starting point [5].

Adult cartilage tissue is avascular in nature. Moreover, the microenvironment of cartilage is hypoxic with oxygen tension as low as 1 %. Therefore, chondrocytes are physiologically exposed to severe hypoxia in vivo [9, 10] and it is well documented in human osteochondral allografts that a high percentage of chondrocytes remained viable after more than 1 month when stored at 4 °C under the optimal conditions of tissue banks [11–13]. This is the reason why cartilage becomes an attractive subject to investigate as a potential new biological method for the estimation of PMI. According to the literature, several authors focused their studies on analyzing the chondrocytes in the determination of PMI - Lackowsky et al. conducted a study *in corpore* [14], and Alibegović et al. conducted in vitro studies [15, 16]. The conclusions of both studies, *in corpore* and in vitro, were that the number of viable chondrocytes depends on the length of the PMI as well as the ambient temperature.

Considering the limitations of the previous studies such as unknown environmental factors and a small, heterogeneous group by donor’s sex and age in the *in corpore* study and laboratory conditions in the in vitro study, we designed an *in corpore* study in controlled environment conditions.

The main aim of this study was to investigate the dynamics of the decrease in the fraction of viable chondrocytes from successfully obtained knee cartilage samples at various times after death. We used knee cartilage since previous studies have shown that it is the most reliable source of cartilage [16–18]. We harvested osteochondral cylinders from the lateral condyles as they are the least affected part of the knee from degenerative changes [19].

The use of chondrocyte viability assays for purposes of estimation of PMI demands a method with good cost/time performance. Therefore, the other goal of the study was to compare different methods for measuring chondrocyte viability and determine the most appropriate one for PMI estimation. In earlier studies, chondrocyte viability was measured either by a confocal laser scanning microscope (CLSM) [13–16] or a cell viability analyzer (CVA), where CLSM provided slightly superior reliability over CVA [16]. CLSM is not standard equipment in forensic medicine institutes. Due to this, the need for a more convenient assay instrument still exists. Flow cytometry (FC) is a conventional technology for cell analysis. It provides a multi-parametric analysis of single-cells as they move past a laser, producing both scattered and fluorescent light signals that are then analyzed by sophisticated software [20–22]. CVA is an instrument currently employed in viable cell counts at many laboratories worldwide. It is a cost-effective method, with fully automatized sample preparation, using a digital camera to obtain images and the analyses are performed through specialized software that requires minimal user involvement [23]. Regarding the aforementioned techniques, we chose to use the CVA and FC for chondrocyte viability assay in our study.

## Materials and methods

The protocol followed the requirements of the Slovenian National Medical Ethics Committee (Permit No. 120–379/2019/8). Subject materials utilized were osteochondral cylinders (OCC) obtained from the lateral condyle of the femur from 42 donors. The donors were deceased individuals from the Ljubljana region who had no family members or other close people to take care of their respective burials. They died either of natural causes (pneumonia, cardiac failure, malignant tumors etc.) or of violent incidents (suspension, severe trauma etc.) at the known time. Per the standard protocol in such cases, the bodies of the individuals are transferred to the central cemetery in Ljubljana, where they await cremation at the request of state services. This occurs after all administrative duties are fulfilled, which could last from several days to months.

After the individuals’ bodies were transferred from the place where they died to the cemetery, between the first or second postmortem day, they were kept in refrigerators set at temperatures 8 ± 2 °C. The donors had no medical history of any knee joint pathology or a systemic illness that could result in cartilage deterioration. We harvested only macroscopically intact cartilage — ICRS grade 0 [24]. Cartilage samples were obtained in the morgue, at room temperature (23 ± 2 °C). At each sampling, 4 cylinders of cartilage with underlying bone measuring 6 × 20 mm were procured with a biopsy needle (Osteochondral Autograft Transfer System, Arthrex, Naples, Florida, US). Every OCC was immediately stored in a 2-mL tube with DMEM (Dulbecco’s Modified Eagle Medium, Merck, Rahwayu, New Jersey, US) supplemented with 5 µg/mL of vancomycin, and 100 µg/mL of gentamicin, and 1 µg/mL of amphotericin B (all produced by Gibco, Paisley, UK). The first sample was taken from the right knee, the second from the left knee, the third from the right knee again, and so on. Following samples were taken weekly, but not always on the same day of the week, depending on the occupancy of the laboratories in which the individual analyses were made continuously one after the other.

### Preparation of the single-cell suspension

**Fig. 1:**
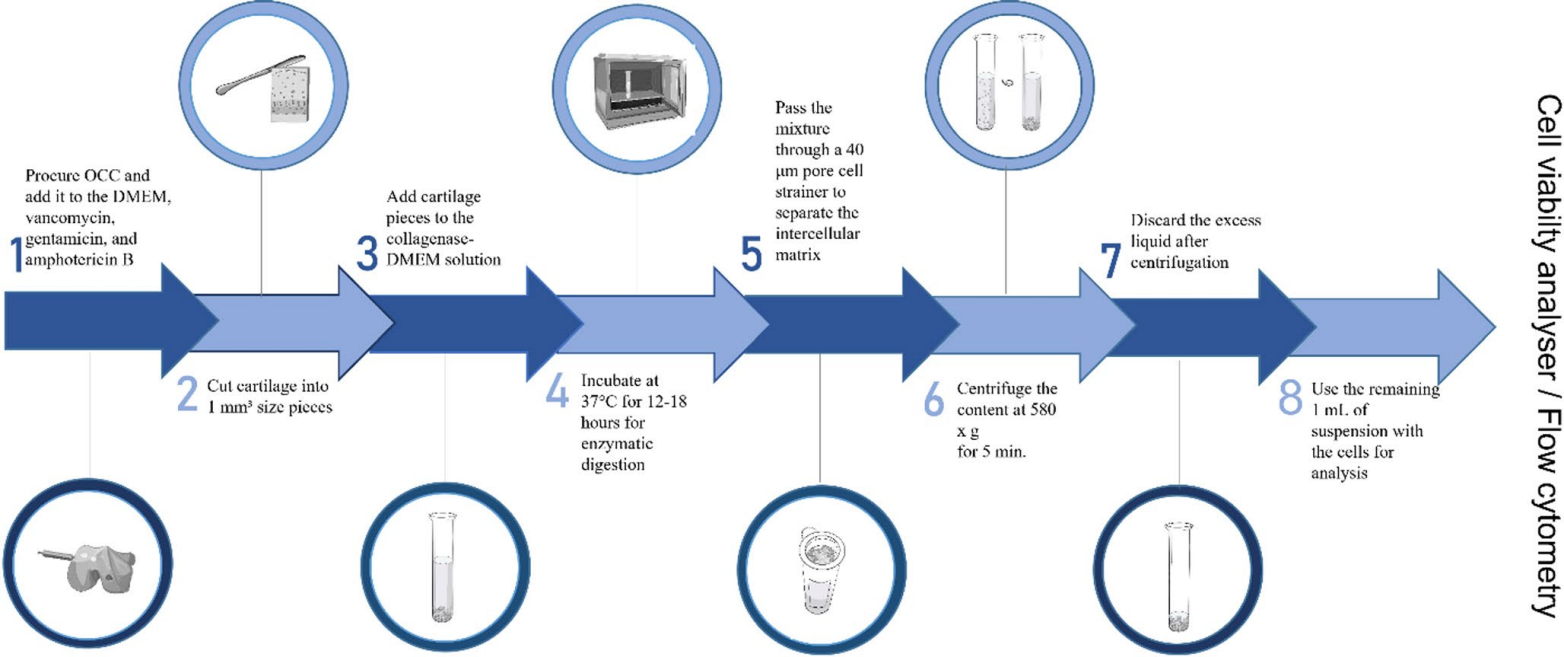
Flowchart of a preparation of single-cell suspension

On harvest day, 30 to 90 min after procuring OCC, preparation of the single-cell suspension was taken according to protocols described by Reichard and Asosingh [25]. On the lid of the Petri dish, four cylinders of cartilage were separated from the bone and cut into small pieces, approximately 1 mm3 in size with a scalpel blade. In the second tube, 13–15 mg of collagenase (C5138, Sigma-Aldrich^®^ Brand, Burlington, Massachusetts, US) was mixed with 13–15 mL of DMEM (mg of collagenase = mL of DMEM). Pieces of cartilage were placed in 10 mL of DMEM and collagenase solution for enzymatic digestion. The cartilage slices were incubated at 37 °C for 12–18 h. The digested cartilage content was washed through a cell strainer with 40 μm pores to separate the intercellular matrix. Additionally, the walls of the tube containing the digested cartilage content were washed with a phosphate buffer and poured over the cell strainer. Cells with residual fluid were centrifuged at 580 x g for 5 min. Following centrifugation, excess liquid was discarded and about 1 mL of suspension, supernatant with cells, remained each time. Preparation of the single-cell suspension process flow diagram is shown in Fig. 1.

### Chondrocytes viability assay

Day after harvest, obtained single-cell suspensions were taken at the Slovenian Institute for Transfusion Medicine and the fraction of viable chondrocytes was determined using CVA and FC.

### Automatic cell viability analyzer

For this method, we used 0.5 mL of a prepared single-cell suspension in a cuvette compatible with a carrousel of CVA (Vi-Cell XR, Beckman Coulter, Brea, CA, US) which automatically adds trypan blue included in the kit for automatic dyeing. Viable cells resist dye passage through the membrane; therefore, only dead cells are marked intensively blue. The parameters for chondrocytes were customized according to the producer (Table 1).

**Table 1:** Customized parameters for Vi-CELL XR Beckman Coulter CVA

Minimum Diameter (microns)	10
Maximum Diameter (microns)	25
Cell Brightness (%)	75
Cell Sharpness	100
Viable Cell Spot Brightness (%)	85
Viable Cell Spot Area (%)	10
Minimum Circularity	0
Decluster Degree	High
Aspirate Cycles	1
Number of Images	50
Trypan Blue Mixing Cycles	3

### Flow cytometry

The single-cell suspension for the FC was centrifuged again (Centrifuge 5415D, Eppendorf, Hamburg, Germany) at 400 x g for 5 min. The supernatant was discarded and the pellet was re-suspended in DPBS (D8537, Sigma-Aldrich^®^ Brand, Burlington, Massachusetts, United States). The isolated cells were stained with the Reddot™1 (1X; Biotium, Fremont, United States) and 7-AAD (7-aminoactinomycin D) viability dye (Miltenyi Biotec, Bergisch Gladbach, Germany) and analyzed with a flow cytometer MACSQuant 10 (Miltenyi Biotec, Bergisch Gladbach, Germany, with software MACSQuantify 2.13.0, Miltenyi Biotec GmbH, Bergisch Gladbach, Germany). The appropriate FC controls were applied throughout and included compensation, time gating, fluorescence minus one (FMO) control and doublet exclusion. With each batch of samples, we also analyzed one control sample with chondrocytes of a person who died within 72 h prior to harvest. These control samples were used as a reference for setting up accurate gates. We recorded 30,000 RedDot™1 positive cells per sample. The results were marked as a percentage of nuclear cells dyed with RedDot™1 (FCN) and a percentage of live cells dyed with 7-AAD inside the RedDot™1 positive cell population (FCC).

### Statistical methods

Data are described as median, interquartile range, and range (minimum to maximum) for continuous variables and frequency and percentage for categorical/discrete variables.

First, the association between the cell viability (fraction) determined by different methods (CVA, FCN, and FCC) and time postmortem (measured in days) adjusted by sex and age was verified using beta mixed effects models with logit link. The association between viability (as the dependent variable) and time postmortem was modeled using a natural spline with 3 degrees of freedom (2 knots set at the 33rd and the 66th centile of time postmortem), setting the boundary knots to 40; this was done as there were very few observations outside this range. Random intercept and random slope for time postmortem by patient ID were included to account for repeated measures. The estimated association between the cell viability and time postmortem is displayed graphically, showing the population-averaged predictions (setting sex and age to their respective mode and median) with 95 % prediction intervals.

Second, to evaluate the predictive value of CVA, FCN, and FCC for predicting PMI, Poisson mixed models with a log link were fitted to the data. Three models were fitted, using either CVA, FCN, or FCC as the independent variable and PMI as the dependent variable; random intercept and random slope for the dependent variable (CVA, FCN, or FCC) by donor ID were included to account for repeated measures. A non-linear association was modeled using a natural spline with 3 degrees of freedom (2 knots set at the 33rd and the 66th centile of CVA, FCN, or FCC). The predictive value of the 3 fitted models was then assessed as follows. The model was fitted on the dataset where one subject was omitted. For the excluded subject, the PMI was predicted using the fitted model (where this subject was not included when estimating the model’s parameters). The process was repeated for each subject, thus obtaining the predictions of PMI for each subject. Root mean squared error (RMSE) and mean absolute error (MAE) were then calculated as follows

$$\:RMSE=\sqrt{\frac{1}{N}{\sum\:}_{i=1}^{n}\sum\:_{j=1}^{{n}_{i}}{({y}_{ij}-{\widehat{y}}_{ij})}^{2}},$$

$$\:MAE=\frac{1}{N}{\sum\:}_{i=1}^{n}\sum\:_{j=1}^{{n}_{i}}|{y}_{ij}-{\widehat{y}}_{ij}|,$$

where $\:N={\sum\:}_{i=1}^{n}{n}_{i}$ is is the total number of observations, $\:{y}_{ij}$ is PMI for individual *i* at the *j*th measurement and ${\hat y_{ij}}$ is the predicted PMI for individual *i* at the *j*th measurement,

where $\:{x}_{ij}^{T}$ is the *j*th row of the fixed effects design matrix $\:{X}_{i}$ and ${\hat \beta ^{ - i}}$is the vector of the estimated fixed effects omitting subject *i*. The results for the models fitted on all subjects are displayed graphically, showing the population-averaged predictions with 95 % prediction intervals.

The marginal r-squared value, which measures the fraction of variability explained by the fixed effects, was calculated for each model as proposed by Nakagawa et al. [26].

R language for statistical computing (R version 4.0.5, cite R Core Team) was used for the analysis [27]. R function glmmTMB from R package glmmTMB was used to fit the model [28, 29]. R function r2 from R package performance was used to calculate marginal r-squared [30]. The statistical significance was set to 5 %.

## Results

**Fig. 2:**
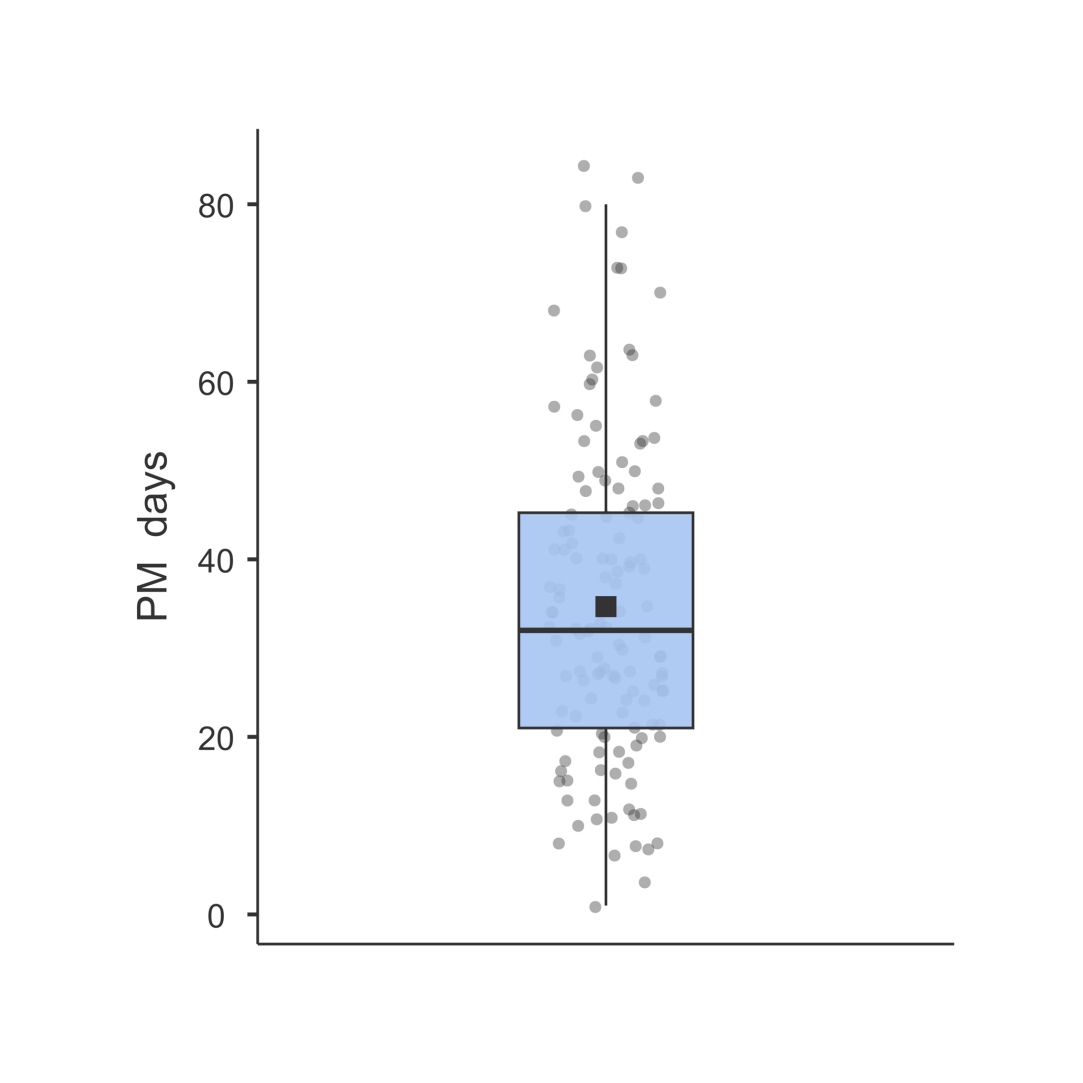
Density of cartilage sampling in the period from 1 to 83 days after death. The blue box represents the interquartile range (IQR), which contains the middle 50 % of the data. The black square inside the box is the mean of the data

For this study, we harvested cartilage from 42 donors. Of these 42 donors, 7 were excluded due to technical issues, therefore, we analyzed OCC from 35 donors (28 males and 7 females), aged 44 to 90 years old, in the period ranging from 4 to 83 days after death (Table 2). Osteochondral cylinders were obtained weekly, until the moment of cremation of the body. Three donors were excluded because the bodies had been frozen due to refrigerator technical problems. Furthermore, DMEM that was standardly used in our research was not available during the COVID-19 pandemic period and consequently, we used a solution with similar ingredients which was not appropriate. Therefore, we analyzed 35 donors, with a total number of 131 samples; 126 by CVA and 128 by FC. Due to a reduced volume of work in our laboratories during the COVID-19 pandemic lockdown or absence of staff due to sick leave, as they performed only urgent routine analyses in this situation, some samples were only analyzed using CVA or FC, resulting in 126 and 128 observations that could be analyzed, respectively. The largest number of analyzed samples was during the period around 20–40 days after death (Fig. 2).

**Table 2:** Characteristics of the studied sample (*n* = 35). The data are median (IQR) and range (from minimum to maximum) for continuous variables and frequency ( %) for categorical (discrete) variables

	Median/frequency (IQR/ %)	Range (min to max)
Age (years)	74 (62–80)	44 to 90
Sex		
Male	27 (77 %)	
Female	8 (23 %)	
Number of measurements		
1	35 (26.7 %)	
2	27 (20.6 %)	
3	25 (19.1 %)	
4	20 (15.3 %)	
5	10 (7.6 %)	
6	7 (5.3 %)	
7	4 (3.1 %)	
8	3 (2.3 %)	
Start of follow-up PM (days)	15 (9-25.5)	1 to 53
End of follow-up PM (days)	38 (25.5–49.5)	4 to 83
Follow-up time (days)	19 (7–27)	0^+^ to 56

Note: IQR– interquartile range; min– minimum; max– maximum; PM– postmortem; ^+^applies to 7 individuals with a single measurement

The age of donors at the time of death ranged from 44 to 90 years (median 74 years, interquartile range (IQR) from 62 to 80 years). The majority of donors were male (77 %). The number of measurements for each donor ranged from 1 to 8 (median 3, IQR from 1 to 4). The median follow-up time was 19 days (IQR from 7 to 27 days). The first measurement was taken after 1 to 53 days postmortem (median 15 days, IQR from 9 to 25.5 days) and the last from 4 to 83 days postmortem (median 38 days, IQR from 25.5 to 49.5 days). Results are shown in Table 2.

**Fig. 3:**
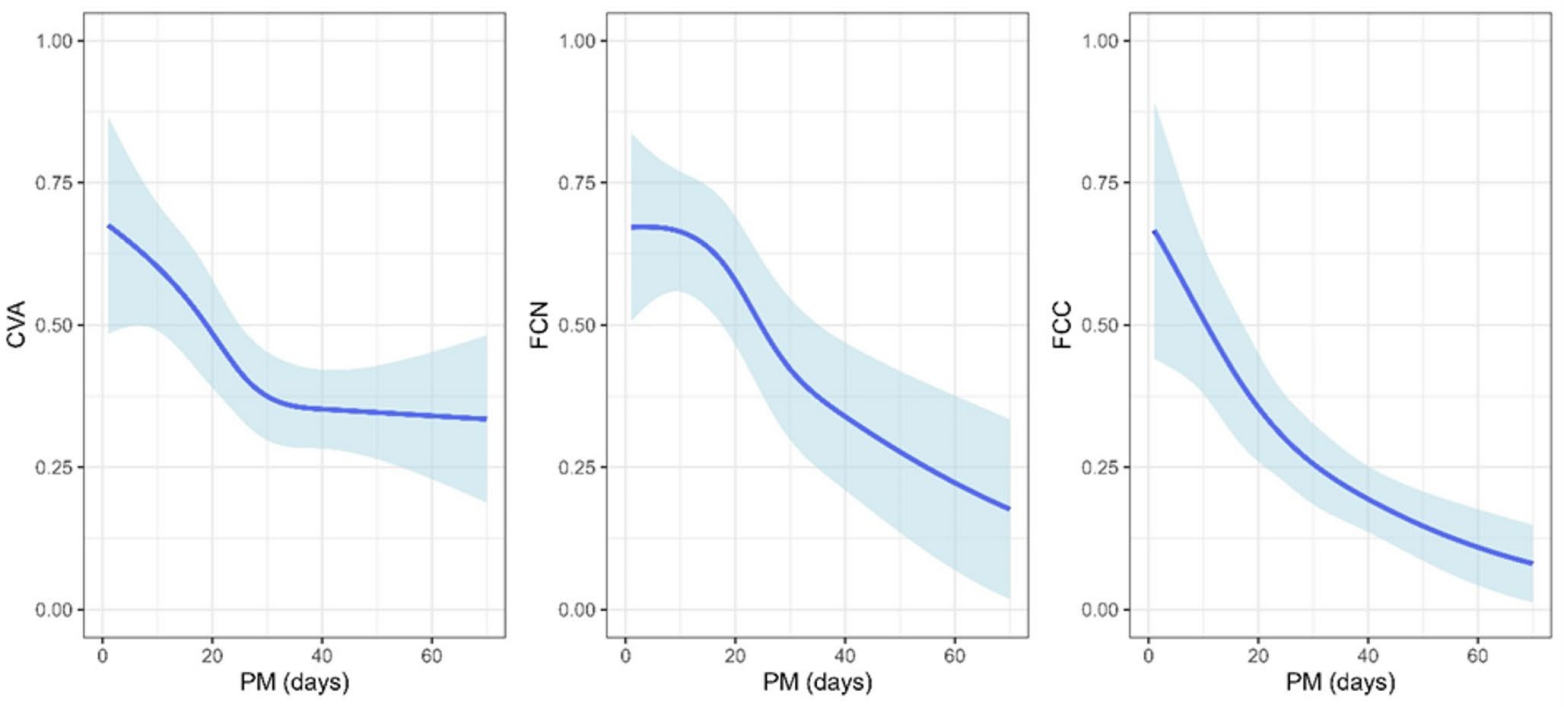
Association between the fraction of the viable cells and PMI showing population-averaged predictions setting sex and age to their respective mode and median (solid blue line) with 95 % prediction intervals (light-blue area surrounding solid blue line) determined by different methods

A statistically significant non-linear association between the cell viability (as measured by CVA, FCN, and FCC) and time postmortem adjusting for sex and age was observed (Table 3; Fig. 3) with the cell viability decreasing as the time postmortem was increasing, with the rate of decrease slowing down (FCN and FCC) or plateauing (CVA) with increasing time postmortem. No association with sex was observed for CVA, FCN, and FCC.

**Table 3:** Estimated fixed effects with standard errors, *p*-values, and 95 % confidence intervals for estimating the association between CVA, FCN, and FCC as dependent variables and time postmortem (PM) modeled as a non-linear association by using a natural spline with 2 knots set at the 33rd and the 66th centile respectively, sex (reference category: males) and age as independent variables

	estimate (SE)	*p*-value	exp(estimate) [95 % CI]
**CVA** (AIC=-112.43, marginal R^2^ = 0.22)			
(Intercept)	-0.94 (0.87)	0.278	0.39 [0.07 to 2.14]
PM		**< 0.001**	
spline1	-1.15 (0.30)	< 0.001	0.32 [0.18 to 0.56]
spline2	-1.75 (0.85)	0.039	0.17 [0.03 to 0.92]
spline3	-1.08 (0.23)	< 0.001	0.34 [0.22 to 0.53]
Sex[female]	0.05 (0.31)	0.863	1.05 [0.57 to 1.94]
Age	0.02 (0.01)	**0.042**	1.02 [1.00 to 1.04]
**FCN** (AIC=-129.57, marginal R^2^ = 0.34)			
(Intercept)	0.30 (1.09)	0.782	1.35 [0.16 to 11.39]
PM		**< 0.001**	
spline1	-1.05 (0.28)	< 0.001	0.35 [0.20 to 0.61]
spline2	-1.25 (0.74)	0.094	0.29 [0.07 to 1.23]
spline3	-1.39 (0.26)	< 0.001	0.25 [0.15 to 0.42]
Sex[female]	0.13 (0.43)	0.757	1.14 [0.49 to 2.65]
Age	0.01 (0.01)	0.709	1.01 [0.98 to 1.03]
**FCC** (AIC=-127.87, marginal R^2^ = 0.34)			
(Intercept)	1.38 (0.91)	0.127	3.99 [0.68 to 23.58]
PM		**< 0.001**	
spline1	-1.38 (0.36)	< 0.001	0.25 [0.12 to 0.51]
spline2	-2.89 (1.00)	0.004	0.06 [0.01 to 0.40]
spline3	-1.53 (0.27)	< 0.001	0.22 [0.13 to 0.37]
Sex[female]	-0.50 (0.34)	0.138	0.60 [0.31 to 1.17]
Age	-0.01 (0.01)	0.429	0.99 [0.97 to 1.01]

Note: estimate– the estimated regression coefficient for the fixed effect; exp(estimate)– exponentiated estimate of the regression coefficient for the fixed effect; CI– confidence interval; spline1, spline2, spline3– B-spline bases for a natural spline; SE– standard error; AIC– Akaike information criterion

**Fig. 4:**
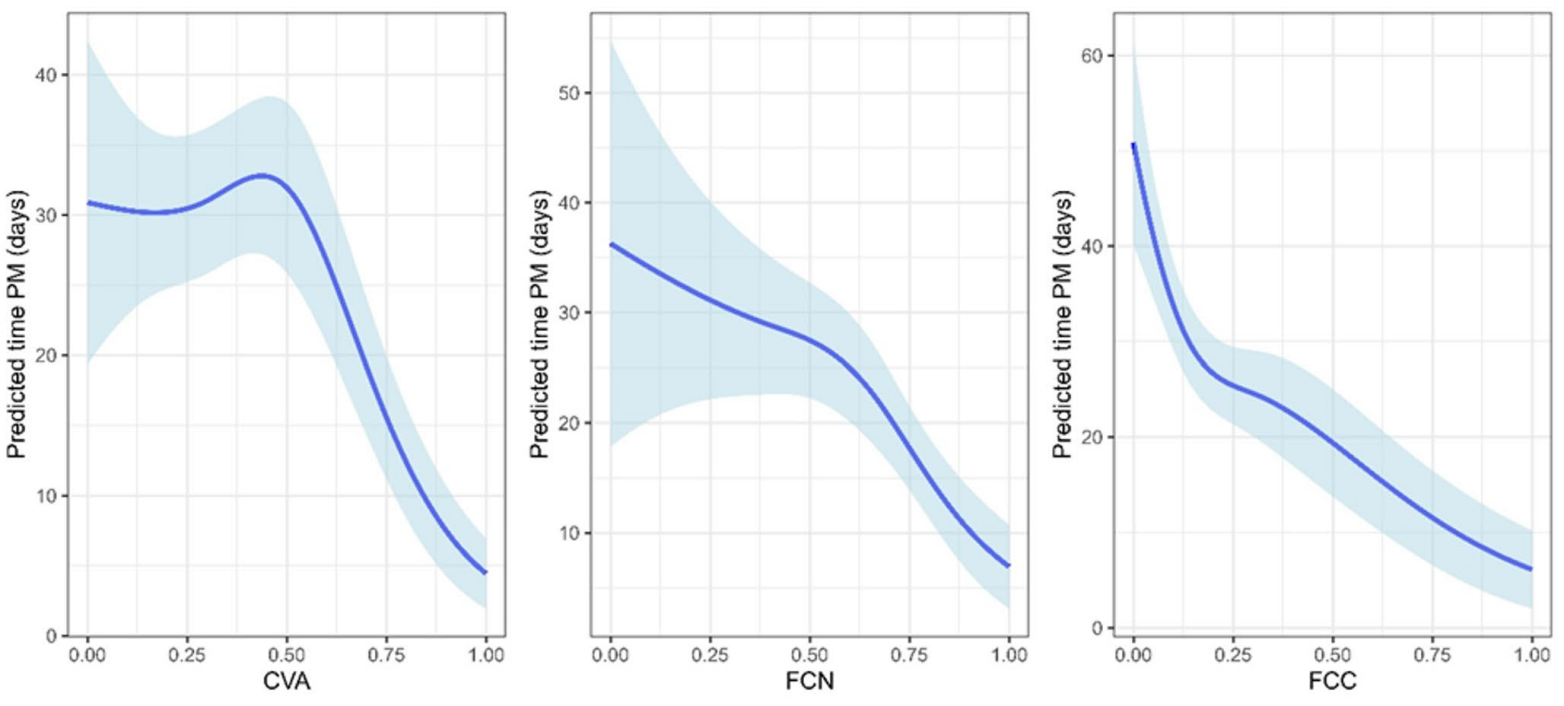
The population-averaged time postmortem predictions (solid blue line) and 95 % prediction intervals (light-blue area) based on CVA, FCN, or FCC

A separate comparison of the CVA’s, FCC’s and FCN’s ability to predict time postmortem showed that FCC is the most promising method in terms of RMSE and MAE, followed by CVA and then FCN (Table 4, see also Fig. 4).

**Table 4:** Estimated fixed effects with standard errors, *p*-values, and 95 % confidence intervals for estimating the association between time postmortem as the dependent variable and CVA, FCN, and FCC as independent variables modeled in separate models as a non-linear association by using a natural spline with 2 knots set at the 33rd and the 66th centile

	estimate (SE)	*p*-value	exp(estimate) [95 % CI]
**CVA** (AIC = 1072, marginal R^2^ = 0.31, RMSE = 16.0, MAE = 12.2)			
(Intercept)	3.43 (0.18)	< 0.001	30.81 [21.65 to 43.84]
CVA		< 0.001	
spline1	0.26 (0.17)	0.132	1.30 [0.92 to 1.83]
spline2	-0.94 (0.39)	0.017	0.39 [0.18 to 0.84]
spline3	-1.62 (0.26)	< 0.001	0.20 [0.12 to 0.33]
**FCN** (AIC = 1101, marginal R^2^ = 0.09, RMSE = 18.3, MAE = 13.9)			
(Intercept)	3.59 (0.26)	< 0.001	36.27 [21.83 to 60.26]
FCN		< 0.001	
spline1	-0.26 (0.25)	0.302	0.77 [0.47 to 1.26]
spline2	-1.17 (0.47)	0.014	0.31 [0.12 to 0.79]
spline3	-1.15 (0.24)	< 0.001	0.32 [0.20 to 0.50]
**FCC** (AIC = 1046, marginal R^2^ = 0.55, RMSE = 15.6, MAE = 11.8)			
(Intercept)	3.90 (0.10)	< 0.001	49.57 [40.48 to 60.69]
FCC		< 0.001	
spline1	-0.48 (0.19)	0.015	0.62 [0.42 to 0.91]
spline2	-1.98 (0.29)	< 0.001	0.14 [0.08 to 0.25]
spline3	-1.68 (0.32)	< 0.001	0.19 [0.10 to 0.35]

Note: estimate– the estimated regression coefficient for the fixed effect; exp(estimate)– exponentiated estimate of the regression coefficient for the fixed effect; CI– confidence interval; spline1, spline2, spline3– B-spline bases for a natural spline; SE– standard error; AIC– Akaike information criterion; RMSE– root mean squared error; MAE– mean absolute error

## Discussion

Accurate estimation of PMI is challenging, especially when dealing with cadavers in advanced stages of decomposition [6]. The decomposition process affects the body’s external appearance, making it difficult to estimate even an approximate PMI. Additionally, factors such as temperature, humidity, and the presence of insects and scavengers can accelerate or slow down the decomposition process, further complicating the estimation of PMI [31, 32]. According to this knowledge, we designed an *in corpore* study under controlled ambient conditions with the body protected from insects and animals involved in decomposition. The main goal of this study was to determine the dynamics of the decrease in the fraction of viable chondrocytes from the knee of a deceased person. We assumed that regularity in this dynamic might place cartilage as a useful tool to establish a new method for the estimation of PMI. Previous pilot studies showed that knee cartilage is the most reliable source of cartilage because it is not affected by rapid decomposition, especially between temperatures around 11 °C and room temperature [15]. Other cartilaginous tissues such as the nasal septum, thyroid cartilage, larynx, trachea, spine, ribs, bronchi, shoulders and hips are expected to be more rapidly affected by decomposition due to their close contact to the commensal bacteria reservoir [16].

Analyzing the cartilage form 35 donors, with 131 samples, we confirmed earlier conclusions about chondrocytes’ ability to live a long period after the death of a host [11–16, 33]. We have observed that chondrocytes from knee cartilage can be found alive after more than two months (Figs. 3 and 4).

Furthermore, our study on whole bodies in the late PMI showed that the fraction of living chondrocytes decreased over time using all methods (CVA, FCN, FCC), which supports the conclusions from previous studies [11–16, 33]. Although fraction of viable chondrocyte’s decreased postmortem, we observed that confident interval around these estimated non-linear association was wide, even in a controlled environmental condition.

Given the relatively small number of measurements, there was considerable uncertainty in the estimation of viability curves, particularly in the initial and final weeks of our study, as can be seen from the light blue areas presented in the figures (Figs. 3 and 4).

Therefore, an extensive study with a larger number of donors and identification of potential factors that are associated with the individual’s variability, such as chronic and prior to death diseases, medications, body mass index, lifestyle etc., might narrow the confident interval in order to create a usable method for the estimation of PMI based on a chondrocyte’s viability.

Based on results from this study, we designed predictive curves for each assay, with 95 % prediction interval, to show how this method could be used in everyday practice (Fig. 4); for an individual with e.g. FCC = 0.75, the predicted PMI is 6 to 17 days, for an individual with FCC = 0.5, the predicted PMI is 13 to 26 days, while for an individual with FCC = 0.25, the predicted PMI is 21 to 30 days.

To determine the fraction of live chondrocytes we used two instruments; CVA and FC. CVA provides automatic and cost/time-effective viable-cell counting, using a trypan blue stain. Since the dye penetrates through damaged membranes of cells, the cells that become stained blue are regarded as non-viable, whereas non-stained cells are assumed to be intact and are considered viable [34].

Flow cytometry (FC) is a modern and advanced diagnostic technique that plays a crucial role in the diagnosis, monitoring of therapies, and assessment of various diseases. It is a gold standard for rapid multi-parametric analysis of single-cells in solutions [20, 35]. When analyzing cells from solid tissues, mechanical and enzymatic degradation is needed and the preparation of single-cell suspension is a crucial part of FC to avoid cell death and aggregation. This procedure should yield a cell population with high viability, minimal cell debris or aggregates, and preserved cell surfaces for FC to be effective [16, 25]. Cell viability can be determined by morphological changes or by changes in membrane permeability. According to this, DNA-binding dyes such as 7-AAD are used as dead cell discriminators in daily routines at laboratories worldwide [36]. Disadvantages of this staining can be gathered debris from dead cells in the sample, shaped to look like live cells and in that way misinterpreted. In this study, in order to avoid false results, we used RedDot™1 to identify all chondrocytes with nuclei (FCN) and 7-AAD, to distinguish live/dead chondrocytes (FCC) among them.

According to the results obtained from this study, FCC is the most promising method for predicting the PMI, followed by CVA and FCN. However, the opportunity of analyzing a huge number of samples in a short time with minimal user involvement requirement and low-cost performance [16], support the CVA to be used in further studies/routine work.

Comparing the results of different chondrocyte assays, we noticed that CVA showed a sharp decline of the curve during the first 30 days, while later it plateaued at about 30 % (Fig. 3). The inconsistency of this observation compared to the results obtained by FC and previous studies is likely due to potential CVA technical issues. One of them might be during the preparation of single-cell suspension such as cell debris and cell aggregation and the other might be due to the set device parameters. Even though in this study CVA was set to specific parameters for chondrocytes (Table 1), software capabilities of the instrument still require better adjustment of input parameters to differentiate cells within clusters allowing a more extensive major control for analyzing samples for each cell line [34].

It is well known that aging impairs the ability of cells to maintain metabolic homeostasis, leading to disruption of cartilage and reducing regenerative capacity. These age-related alterations are recognized as major contributors to the development of osteoarthritis and the progressive deterioration of articular cartilage [37]. In this study, we observed no age-related differences in the ratio of live chondrocytes when assessed using the FC method. An exception was noted in the CVA method, where an even higher proportion of live chondrocytes was found in older donors (Table 3). We hypothesize that this unexpected result may be due to the inclusion of only macroscopically intact cartilage into this study, which could account for the preservation of chondrocyte viability in older individuals. It is also important to note that our assessment focused on the ratio of live/dead chondrocytes, rather than the absolute number of viable cells. Although the total number of chondrocytes in healthy cartilage decrease with age, the rate of postmortem chondrocyte degradation may not necessarily be age dependent. High viability detected by CVA in older donors may be due to technical issues rather than to biological differences.

The use of human osteochondral allografts (OCA) has become a vital option for orthopedic surgeons to treat chondral and osteochondral lesions primarily of the knee, as well as lesions in the ankle, hip, and shoulder. Fresh OCA tissues are ideally harvested from healthy donors, with healthy gross articular cartilage, aged 14 to 50 years within 24 h of death [38]– [39]. Although cartilage from older individuals is generally more susceptible to degenerative changes, our findings suggest that, when macroscopically intact, cartilage from elderly donors may still possess adequate chondrocyte viability and could thus be considered as a potential source for OCA transplantation.

Most of the previous studies for the determination of the PMI did not show differences between males and females when calculating PMI [40–46]. In addition, none of the three cell viability assays (CVA, FCN, FCC) used in this study showed significant differences in the fraction of live chondrocytes between males and females which suggests that rapidity of postmortem body changes is not associated with sex.

The ability of chondrocytes to remain viable for extended periods after the death of individuals has attracted the attention of forensic experts in the last few years. In this study, we were able to generate prediction curves with a 95 % confidence interval based on postmortem chondrocyte viability, but the confidence interval was wide in some periods and as such is not currently applicable in daily practice. Larger studies could gather more information about individual differences. Furthermore, it is essential to conduct studies in different post-mortem environmental conditions such as temperature, burial, and precipitation to evaluate the effects of external factors on cellular degradation [47]. Incorporating specific correction factors, like those used in the Henssge nomogram, could contribute to the development of more precise, quantitative methodologies for estimating late PMI based on chondrocytes viability.

## Conclusions

Due to the long survival potential after an individual’s death, chondrocytes are a valuable origin of information that could be used for late PMI determination. Further cartilage biology research as well as larger *in corpore* studies would be helpful for better understanding chondrocytes’ postmortem changes timeline. Even though FCC showed a slightly higher potential in predicting PMI, as was evident by the smallest RMSE and MAE, CVA also showed potential and could be used in further research. Results from this study shown no differences between ages which implicates that the cartilage of elderly donors could also be considered as a source in human OCA transplantation.

## Electronic supplementary material

Below is the link to the electronic supplementary material.

Supplementary Material 1

## Data Availability

All the data and material used in this manuscript are in the possession of authors.
