# Supplementary material for: Significance of chondrocyte viability in postmortem interval assessments and chondrocyte viability assay
Source: Int J Legal Med. 2025 Jul 16;139(6):2763–73. doi: 10.1007/s00414-025-03549-4 (PMC12532684; doi:10.1007/s00414-025-03549-4)
Supplement: Supplementary file 1 — Supplementary Material 1 [file 414_2025_3549_MOESM1_ESM.docx]

Contribution Details

|  | Anita Galić Mihić | Davor Mayer | Katerina Jazbec Gradišar | Elvira Maličev | Rok Blagus | Pero Hrabač | Armin Alibegović |
| --- | --- | --- | --- | --- | --- | --- | --- |
| Concepts | + |  |  |  |  |  | + |
| Design | + | + | + | + |  |  | + |
| Definition of intellectual content | + | + | + | + | + | + | + |
| Literature search | + | + | + | + | + | + | + |
| Clinical studies |  |  |  |  |  |  |  |
| Experimental studies |  |  |  |  |  |  |  |
| Data acquisition | + | + | + | + | + | + | + |
| Data analysis | + |  |  |  | + | + | + |
| Statistical analysis |  |  |  |  | + | + |  |
| Manuscript preparation | + | + | + | + | + |  | + |
| Manuscript editing | + |  |  |  |  |  | + |
| Manuscript review | + |  | + |  | + |  | + |
| Guarantor | + |  |  |  |  |  | + |
